# Nano-clays as Potential Pseudo-antibodies for COVID-19

**DOI:** 10.1186/s11671-020-03403-z

**Published:** 2020-08-28

**Authors:** Sahel N. Abduljauwad, Taimur Habib, Habib-ur-Rehman Ahmed

**Affiliations:** 1 Civil & Environmental Engineering department, King Fahd University of Petroleum & Minerals (KFUPM), Dhahran, Saudi Arabia; 2 Royal College of Surgeons in Ireland (RCSI), Bahrain campus, Busaiteen, Bahrain; 3 Engineering & Research International (ERI), Riyadh, Saudi Arabia

## Abstract

Despite several efforts, the development of an effective vaccine for COVID-19 may take a much longer time. Traditional/natural medicine, already experienced by humans, could be an earlier solution. Considering the research team’s experience in using nano-clays as high-affinity material for cancer metastasis, melanoma treatment, and bone regeneration, we propose to use these nano-clays for the prevention/treatment of COVID-19. Owing to high affinity, nano-clays would capture the viruses before the latter get engaged with human hACE2. In this study, molecular-level simulations and modeling of the interaction of coronavirus spike and hACE2 proteins were performed with and without nano-clays. The results showed a very high level of affinity/cohesiveness among SARS-CoV-2 spike and nano-clays as compared to the one between the former and hACE2. We premise that these nano-clays since already being used as drug carriers could also be injected as “clays-alone” medicine. Recommendations have also been provided for future in vitro and in vivo studies.

## Background

**Fig. 1:**
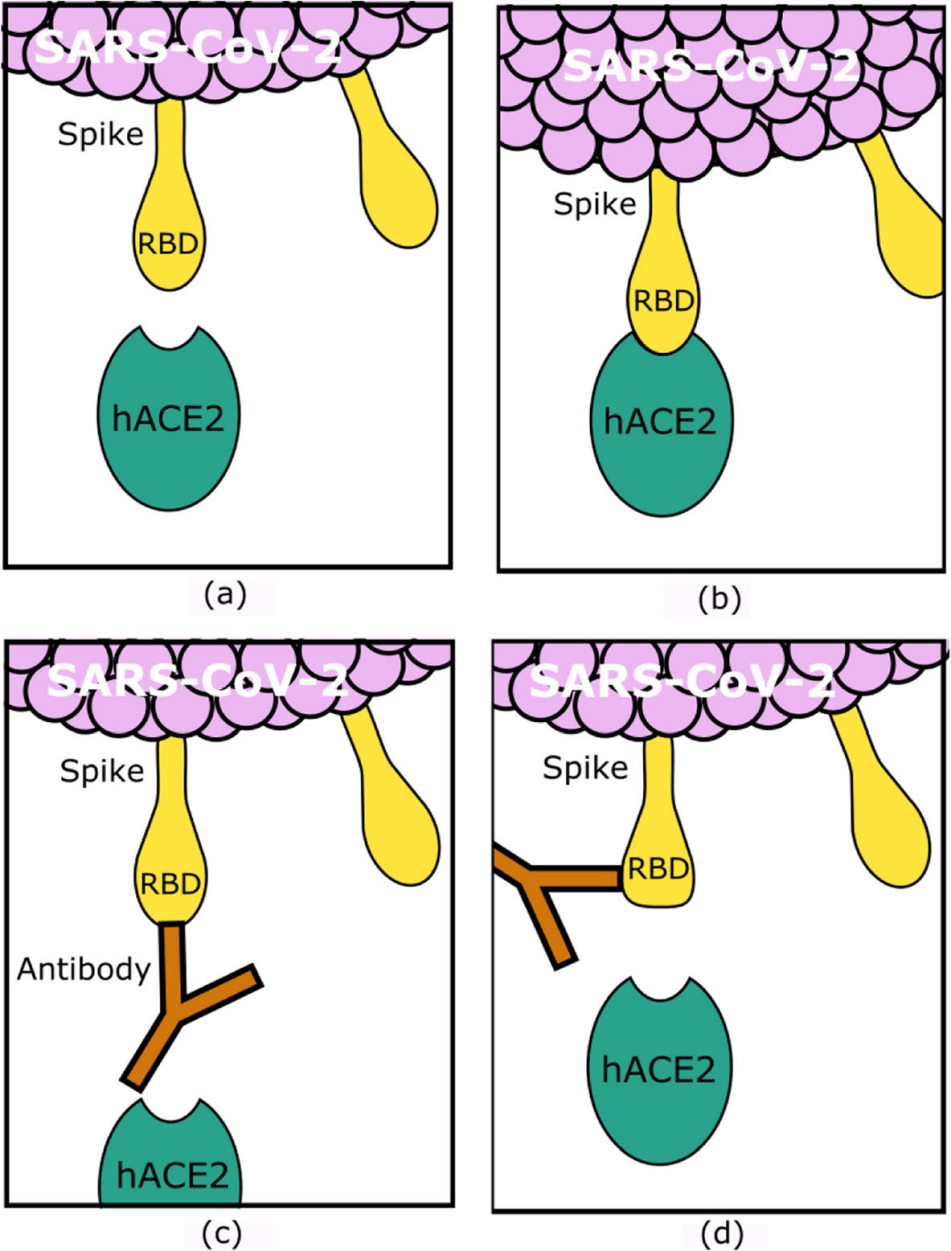
Schematics of the SARS-CoV-2 attack on human hACE2 and the subsequent immune system response. **a**, **b** RBD binding hACE2 without an interference. **c** RBD complexed with the antibody at receptor attachment site hence competing with hACE2. **d** RBD complexed with RBD at a site other than where receptor attaches resulting in the alteration of RBD structure and interruption of lock and key binding of RBD to hACE2

The sudden emergence and rapid spread of novel coronavirus, SARS-CoV-2, have significantly affected the health and lives of human beings in addition to critically affecting the world economy. SARS-CoV-2 spike S binds with high affinity to human angiotensin-converting enzyme 2 (hACE2) and uses it as an entry receptor to invade target cells (Fig. 1a, b) [1]. The virus-surface spike protein mediates coronavirus entry into host cells. SARS-CoV-2 spike protein contains a receptor-binding domain (RBD) that recognizes explicitly as its receptor hACE2 [2, 3]. The surface of hACE2 contains two virus-binding hotspots that are critical for SARS-CoV-2 S binding. Several naturally selected mutations in SARS-CoV-2 RBD surround these hotspots and regulate the infectivity, pathogenesis, and cross-species and human-to-human transmissions of SARS-CoV-2 [2, 4, 5].

At present, there are no clinically approved vaccines or drugs that specifically target SARS-CoV-2. Following the real protocol of developing a vaccine, it may take much longer time to come up with an effective vaccine. There is a lot of interest in the development of therapeutic antibodies against SARS-CoV-2. Despite many efforts however, these antibodies have not yet been discovered [6] except in a few trials [7]. One trial showed the potent neutralization of SARS-CoV-2 by binding to the RBD of its S glycoprotein [8]. In this trial [8], antibody cocktails, a mixture of different antibodies is recommended due to the increased neutralization effect it has on SARS-CoV-2. However, use of antibodies in the past from convalescent patients of SARS-CoV to treat SARS-CoV infection has shown adverse reactions in the patients such as Antibody-Dependent Enhancement (ADE) causing increased viral infectivity and other harmful immune responses [7]. Moreover, based on the experience with the vaccine development efforts for SARS-CoV and MERS, chances of the materialization of the efforts being made for SARS-CoV-2 seems quite thin. Therefore, natural/traditional medicines that have a history of safe consumption/ingestion by humans could be considered as one of the treatment options for SARS-CoV-2. Being a natural material and a history of human use/consumption, we suggest “highly charged nano-clays” to be used as coronavirus blockers and inhibitors of the spike-mediated entry into the human cells.

**Fig. 2:**
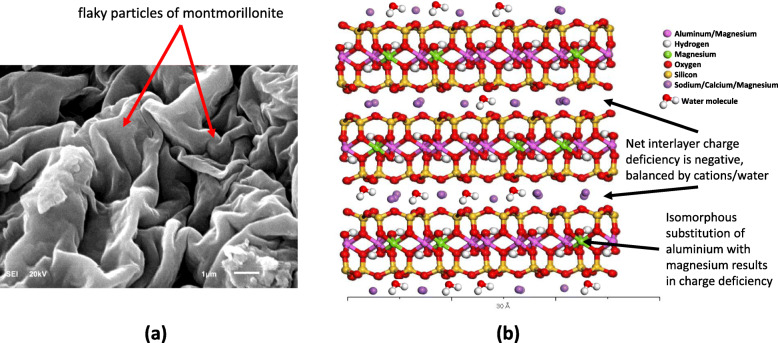
**a** SEM image and **b** the corresponding molecular structure of Na-montmorillonite showing the configuration, isomorphous substitution, charge deficiency, and interlayer cations (from [10])

Nano-clays, nano-sized natural materials originating from minerals of the sedimentary rocks, have got a very high affinity to bacteria and viruses [9]. Due to isomorphous substitution in their molecular structure, these nano-clays exhibit charge deficiency on their surfaces. This charge deficiency on their surfaces is neutralized by the water molecules and the dissolved cations (Fig. 2). The charged structure and large surface area of clay nanoparticles give them an affinity for charged entities, as found on bacterial surfaces and bacterial toxins. Their distinct biomedical properties include high absorption, the ability to engulf microbes, and no toxicity. Each of the electrically active clay minerals has its distinct morphology, characteristics, and interaction behavior. The most studied biomedical application of nano-clays includes serving as carriers and complexes for anticancer drugs such as 5-fluorouracil and trastuzumab [11–17]. They have, therefore, been a potential alternate medicine for several diseases [18–22]. Clay nanoparticles, due to their adhesive nature, have also been used as carriers for sustained-release medicine [15, 23]. Nano-clays have also successfully been used to adsorb and treat bovine rotavirus and bovine coronavirus [24]. Researchers [25] intercalated methotrexate (MTX), an anticancer agent, into the anionic clay to create a nano-hybrid drug. They used the co-precipitation and subsequent hydrothermal methodology to prepare this chemically, structurally, and morphologically well-defined two-dimensional drug-clay nano-hybrid. The researchers [26] discovered that due to the biocompatibility and high loading capacity, bentonite nano-clay could be used for the preparation of the drug-delivery vehicles. In this study, they prepared doxorubicin-bentonite nano-clay complex (DOX-Bent complex) to form a sustained-release drug-delivery system for intra-tumoral chemotherapy of melanoma. As montmorillonite clay is recently being studied to be used as an additive and drug carrier material, these nano-clay composites appeal their use in various dosing form, mainly for controlled release of the drug [27]. The researchers [28] also discovered that nano-clays can be used into recent dual functional drug delivery systems (DDSs) to have efficiency in the drug delivery and so reduce the toxicity of doxorubicin (DOX) that is being used for thyroid cancer treatment. Using a library of 12 single–single type photo cleavable amphiphilic Janus dendrimers, researchers [29] developed a self-assembling light-responsive dendrimersomes vesicle platform. Similar to the nano-clays, surface modified bioactive virus-mimicking organic nano-vesicles from (glyco)dendrimersomes have structural modifications that contribute to manifest SARS-CoV-2 and host pathogenic molecular interactions that help the virus to escape from the human immune system [30].

Through considerable previous research, we developed basic characterization and behavior modeling of the charged clay minerals [31–33], and their applications in the control of cancer metastasis [10], in vitro and in vivo studies on melanoma treatment [34], and the calcium deposition/bone regeneration studies [35]. In a previous study by the authors [35], it was demonstrated that clay nanoparticles had got a high affinity to the charged surfaces. The high attraction affinity of the nano-clays and the increased non-specific adhesion attraction of the cancer cells make nano-clays favorable candidates to control cancer metastasis. In that study, we demonstrated the possible use of two charged clay minerals to control the metastasis of the cancer cells: Na-montmorillonite (SWy-3) and palygorskite (PFl-l). Further to the findings of the authors’ previous research [35] on the use of these nano-clays for the control of cancer metastasis, we also, through in vitro and in vivo studies, established that these nano-clays have inhibitory effects on melanoma cancer cells, mainly on cell proliferation and viability [34]. In these previous studies, in addition to laboratory experiments, molecular-level simulations were also performed on the nano-clay and cells’ interactions. These simulations provided the assessment of the relative level of cohesiveness/affinity in the interactions with and without clay nanoparticles.

Based on all the above experience of the authors on the high-affinity potential of nano-clays, we propose that the nano-clays could be mimicked as antibodies and can thus attract and engulf coronaviruses before they get engaged with human hACE2. This paper is a first step towards establishing this perception through a molecular-level simulation and modeling approach. Based on the results of the molecular-level simulations, an outline of the recommendations for the next phases of in vitro and in vivo research is also provided. As these nano-clays are also successfully being used as medicine carriers, we also premise that they can also be injected/ingested as “clays-alone” medicine, and thus, we have proposed a tentative nano-clays administration methodology for this purpose.

## Materials—Molecules

### Selection and Formulation of SARS-CoV-2 and hACE2

**Fig. 3:**
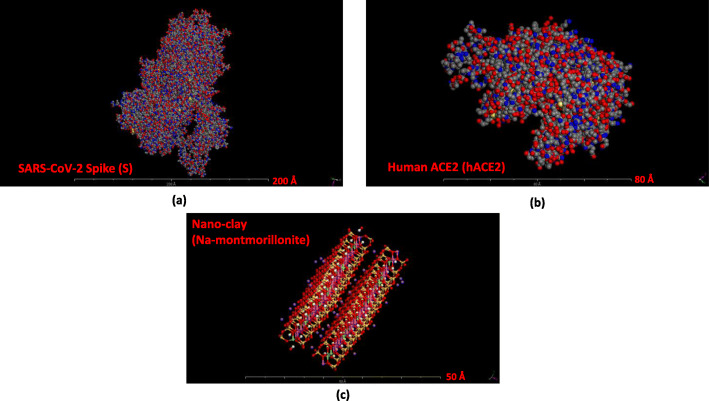
Molecular-level models of **a** SARS-CoV-2 spike, **b** hACE2, and **c** Na-montmorillonite crystallite formulated in Materials Studio software

Molecules of SARS-CoV-2 spike S and hACE2 were acquired from the protein data bank website RCSB [36–38]. The molecular models of SARS-CoV-2 spike S and hACE2 formulated in Materials Studio software [39] are respectively shown in Fig. 3a, b. Before being subject to the simulations, these molecules were charged using the charge equilibration method QEq of the software.

### Selection and Formulation of Nano-clay Crystallite

Na-montmorillonite, one of the most active members of the smectite group of clay minerals, was selected for the study. Na-montmorillonite is a layered phyllosilicate clay smectite (Fig. 2). In the colloidal form, the space between neighboring layers can contain free sodium, calcium, or magnesium cations that are electrostatically attracted to external negatively charged surfaces [40]. In its dry powdered state, Na-montmorillonite exists as equidimensional flakes/sheets with dimensions of approximately 0.5 × 0.5 × 0.001 microns (Fig. 2a). These negative charges on their interlayer surfaces are balanced by the cations. As colloids, the interlayer cations get dissociated from the clay particles and associate themselves with the other negatively charged surfaces. These particles also have positively charged edges due to the presence of the broken bonds at their ends. Morphology and further characteristics of these nano-clays are provided in Table 1, while formulation of their crystallites in Materials Studio software are explained below.

**Table 1:** Summary of physical and chemical characterization of Na-montmorillonite clay (SWy-3) [40]

Source	Chemical formula	Other minerals	Surface area N_2_ (m^2^/g)	CEC (meq/100 g)	Exchangeable cations	Octahedral charge	Tetrahedral charge	Interlayer charge	Zeta potential (ZP), (mV)	γ_s_^-^	Water affinity	Interaction energy, (AB)	Interaction energy (vdW)	Interaction energy (total)	Flocculation/dispersion in water
Crook County, WY USA	(Na,Ca)_0.33_(Al,Mg)_2_(Si_4_O_10_)	5% silica	31.82	76.4	Na, Ca	− 0.53	− 0.02	− 0.55	− 31.9	44.6	Hydrophilic /polar	22,400	− 730	22800	Dispersion

In the software, Na-montmorillonite crystallites were formulated based on fundamental properties such as CEC, exchangeable cations, and interlayer charges (Table 1). The size of the molecular/crystallite size was selected based on the results of the particle size analysis using the dynamic light scattering (DLS) technique [10]. The final form of clay crystallite created in the software is shown in Fig. 3c. After the preparation of these crystallites in the design mode of the software using the inherent properties, these were charged using the charge equilibration method QEq of the software.

## Methods—Molecular-Level Simulations

This part of the study consisted of the simulation and assessment of the interactions of the SARS-CoV-2 spike S with clay crystallites and with hACE2. Although these models may not be the complete replication of the actual in vitro conditions, these have been incorporated with all the essential interactions and are quite well suited for the intended relative and comparative study.

In the software, the sorption and simulations of the formulated configurations of SARS-CoV-2 S, Na-montmorillonite crystallites, and hACE2 were carried out using Monte Carlo (MC) and molecular mechanics (MM) techniques. The enhancement of affinity in all the simulated configurations was assessed in terms of the calculated cohesive energy density (CED)—CED being considered as a measurement of the cohesiveness of the molecular system. Due to the large-sized computations involved in the simulations, these calculations were carried out using the high-performance computing facilities (HPC) at KFUPM, KSA. The overall methodology and the choice of particular methods and the simulation parameters were based on authors’ previous research [41–47], while it is detailed in the subsequent section.

### SARS-CoV-2 Spike (S) Interactions with hACE2 and Clay Crystallites

To simulate the interaction of SARS-CoV-2 S with clay crystallites, various numbers of the crystallites of Na-montmorillonite clay were sorbed on SARS-CoV-2 S model. For these sorption simulations, the Metropolis Monte Carlo method was selected in the Sorption module of the software. In each sorption step, clay crystallites occupy spaces around the spike S model to lower the overall energy of the complex. The required number of crystallites were sorbed in a maximum of 25,000 steps, and then, the energy of the system was minimized using the Forcite module of the software based on the MD principles. The similar sorption process was repeated for the interaction modeling of the SARS-CoV-2 spike molecule with hACE2. In this process, hACE2 molecules were sorbed around the RBD of the spike S of SARS-CoV-2. After the completion of the sorption process, the energy of the formulation was minimized using MD-based module of the software.

The Forcite module of the software incorporating NPT (constant number of particles, pressure, and temperature) ensemble was used for MD simulations with a modified universal force field [41]. The simulations were run for 5 to 30 ps with an interval of 0.5-fs or till a constant volume is obtained. A Berendsen thermostat with a decay constant of 0.1 ps was used to control the temperature during the simulation. During the MD simulations, the assumed temperature was kept constant at 310 K (37 °C) with an atmospheric pressure (100 kPa). A Berendsen barostat with a decay constant of 0.1 ps was used to control the pressure of the system. The Berendsen methodology was considered as the most appropriate for the single crystallites after several trials involving other thermostats and barostats available in the software. In the Monte Carlo method, the parameters for the ratios of exchange, conformer, rotate, translate, and regrow were selected as 0.39, 0.2, 0.2, 0.2, and 0.2 respectively with the corresponding probabilities as 0.39, 0.2, 0.2, 0.2, and 0.2. Amplitudes adapted for rotation and translation were 5° and 1 Å, respectively.

### Cohesive Energy Density (CED) Measurement

In this study, the assessment of the affinity/binding level in the SARS-CoV-2-clay crystallites and SARS-CoV-2-hACE2 complexes was measured through the changes in the CED. After the sorption of clay crystallites and the subsequent performance of molecular dynamics of each of the configurations, the CED was determined using the cohesive energy density option of the Forcite module of the software. The authors have experienced that the CED concept, consisting of the total, van der Waals and electrostatic CEDs, can quite closely explain the various molecular-level processes and interactions and simulate the extent of affinity/binding created among the simulated complexes [41–47]. Quantitatively, CED is defined as the amount of energy needed for the transition of 1 mol of material from the liquid to the gaseous phase. It is also a measure of the mutual affinity/attractiveness of molecules and is expressed both as electrostatic and van der Waals forces, averaged over an NPT ensemble.

In the Forcite module, van der Waals energies were evaluated using atom-based cutoffs. In this method, non-bond interactions are simply calculated to a cutoff distance, and interactions beyond this distance are ignored. To avoid the discontinuities caused by direct cutoffs, most simulations use a switching function to turn off non-bond interactions over a range of distances smoothly. An effective potential is created by multiplying the actual potential by the smoothing function. The choice of the function in the intermediate range is crucial and should be continuously differentiable in this region so that forces can be calculated. In this study, a cubic spline smoothing function was used with a spline width of 1 Å and a cutoff distance of 12.5 Å.

## Results and Discussions

**Fig. 4:**
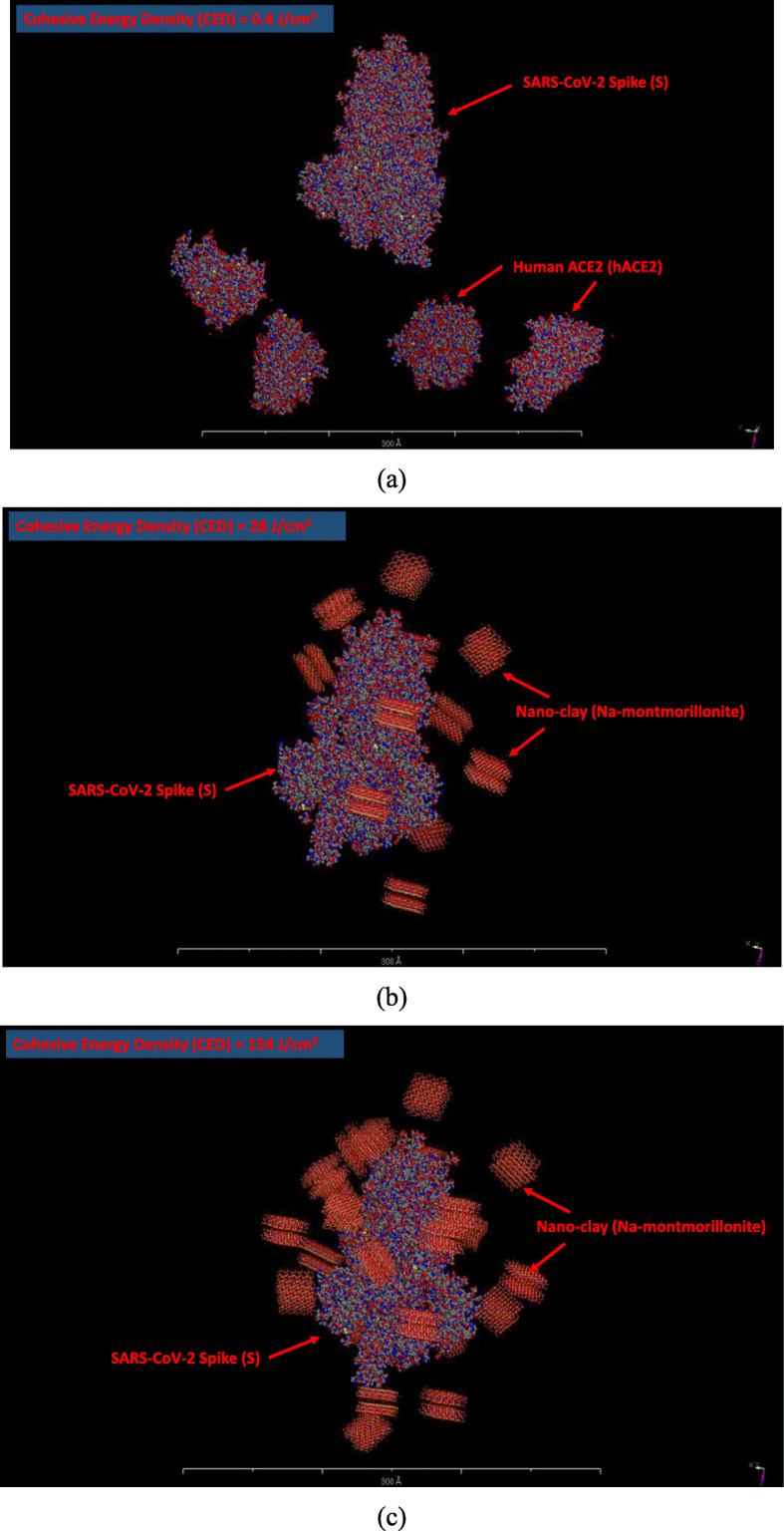
Molecular-level simulation results in Materials Studio Software. **a** SARS-CoV-2 S and hACE2 (CED = 1 J/cm^3^), **b** SARS-CoV-2 S model interacting with twelve crystallites of Na-montmorillonite (CED = 28 J/cm^3^), and **c** SARS-CoV-2 S model interacting with twenty-four crystallites of Na-montmorillonite (CED = 154 J/cm^3^)—obtained using Sorption technique implemented in the software

**Fig. 5:**
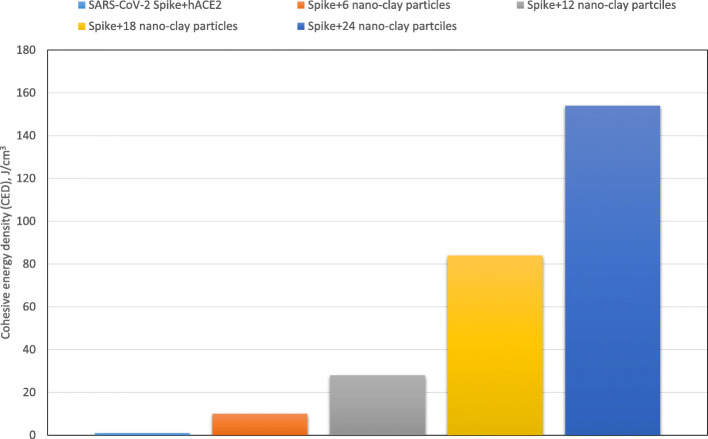
Variation of cohesive energy density (CED) for SARS-CoV-2 S-hACE2 and the complexes of the former with different numbers of Na-montmorillonite crystallites

The final configuration of the SARS-CoV-2 S-hACE2 complex is shown in Fig. 4a, while the complexes between SARS-CoV-2 spike and different numbers of clay Na-montmorillonite crystallites are respectively shown in Fig. 4b, c. For comparison purposes, total CEDs of various proportions/numbers of the clay crystallites on the SARS-CoV-2 spike and the interaction of the later with hACE2 are plotted in Fig. 5.

Based on our experience, we have hypothesized that nano-clays, due to their high adhesive properties, could also act as SARS-CoV-2 inhibitors. They can do it by strongly associating with the spike S present on SARS-CoV-2. The results obtained from the molecular-level simulations of the interactions indicate that due to very high CED between SARS-CoV-2 and the nano-clays as compared to the former and hACE2 (Fig. 5), they could inhibit SARS-CoV-2 from getting engaged with hACE2. Moreover, it could also be concluded from Fig. 5 that the extent of inhibition due to nano-clays is increased in quantity (dosage)-dependent way.

### Nano-clay Interactions with SARS-CoV-2 Spike S

**Fig. 6:**
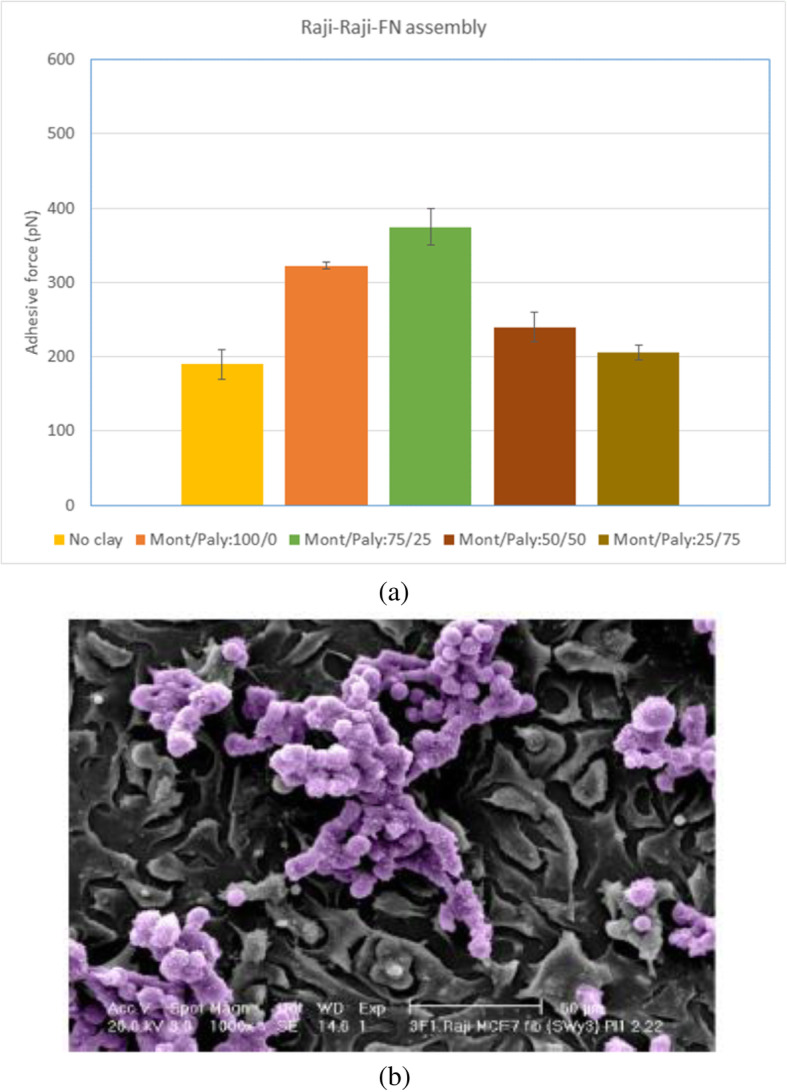
**a** Summary of adhesion force measurements among Raji-Raji-FN assembly using AFM, before and after treatment with various proportions of Na-montmorillonite and palygorskite clay nanoparticles [10]. Error bars represent the variations in the trials. **b** SEM image of the binding of Raji cells and Fibronectin proteins produced by nano-clays

**Fig. 7:**
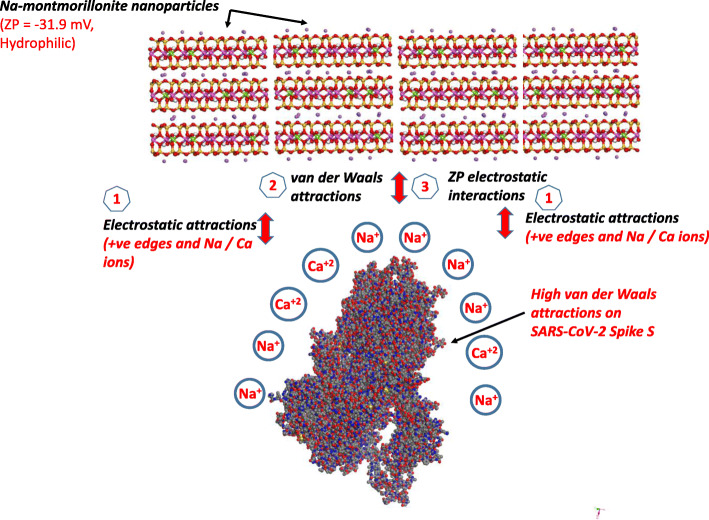
Three possible mechanisms of interactions of montmorillonite nano-clay with the SARS-CoV-2 spike S: (1) Electrostatic attraction among positively charged nanoparticle edges and Na/Ca ions with negatively charged virus surfaces. (2) Van der Waals attractions. (3) ZP electrostatic interactions

Authors, in their earlier research, have demonstrated the role of nano-clays in promoting adhesion among the cancer cells and their microenvironment and hence controlling metastasis [10]. Adhesion measurements of 75/25 mix of Na-montmorillonite and palygorskite showed an increase in adhesion by 100% among cancer cells and the extracellular matrix proteins (Fig. 6a). A corresponding SEM of the nano-clays binding the Raji cells and the fibronectin proteins is shown in Fig. 6b. Sample imaging was performed in SEM mode in an FEI ESEM-FEG XL-30 at the Miller School of Medicine, University of Miami, Florida. Authors also discovered in their previous research that electrostatic, van der Waals, and ZP attractions seem to be dominating in the adhesion processes [10]. We conclude that the same mechanisms would have also facilitated the binding of the adhesive surfaces of the nano-clays to the spike of SARS-COV-2 (Fig. 7). ZP is a measure of the dispersion or flocculation tendency in the colloidal form, including the interactions with the other constituents present in the suspension medium. As a general rule, a zeta potential greater than 30 mV (either positive or negative) indicates dispersion tendency, while a zeta potential of less than 5 mV generally results in agglomeration. Higher dispersion tendencies ZP of the clay nanoparticles used in the study (− 24 to − 32 mV) lead to higher dispersion tendency and hence in the generation of higher surface area amplifying the interactions with the SARS-CoV-2 spike. Although based on their ZP, Na-montmorillonite nanoparticles have hydrophilic nature, they, in the presence of salts, also promote secondary adhesion mechanisms between hydrophobic and hydrophilic surfaces [10]. It should also be noted that these clay nanoparticles have high dispersion tendency due to their hydrophilic nature and relatively higher repulsive acid-base (AB) interactions (Table 1). High dispersion, in turn, results in the generation of high surface area for increasing the attractive interactions. Higher surface areas promote larger attractions due to the van der Waal attractions and the electrostatic forces among oppositely charged surfaces. Besides, although of relatively lesser degree, positively charged edges of Na-montmorillonite particles also get electrically attracted to the spike S.

The results of the molecular-level simulations for the interaction of SARS-CoV-2 spike S with the clay crystallites (Fig. 5) also confirm the above interaction behaviors. It has been observed that the sorption of the clay nanoparticles results in the formation of closely interacting strong van der Waals attraction fields. These van der Waals attraction fields create higher CED of the clay/SARS-CoV-2 configuration. Substantial increase in total CED after the addition of clay crystallites (Fig. 5) is also a testimony of a very high affinity of SARS-CoV-2 with these particles as compared to the affinity of the former with hACE2.

### Nano-clays as Pseudo-antibodies

**Fig. 8:**
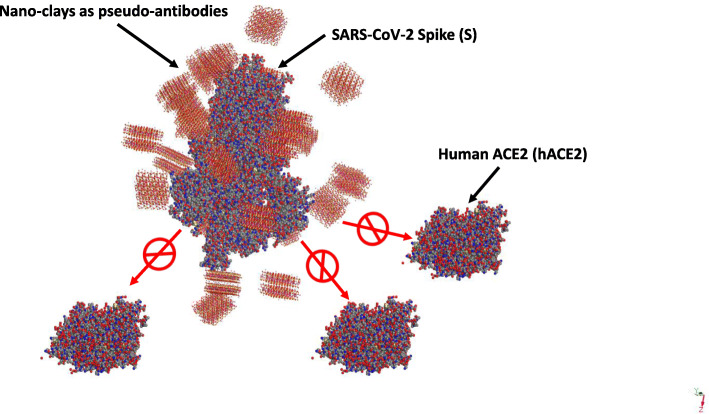
Interaction mechanism of nano-clay particles with SARS-CoV-2 spike S inhibiting the interaction of the later with hACE2

Based on all the current and past research by the authors, establishing the high-affinity potential of nano-clays, we premise that nano-clays could be mimicked as antibodies and can thus attract and engulf coronaviruses before they get engaged with human hACE2. Antibodies are glycoproteins synthesized by plasma cells as part of the adaptive immune response to assist in the clearance of infection from the body. Antibodies aid in infection clearance in multiple ways, such as opsonization of pathogens to facilitate phagocytosis, activation of the complement system, agglutination of microbes, and neutralization of viruses and toxins. When bound to the viral surface proteins, antibodies prevent the entry of the viruses into the cell by preventing the attachment of viruses to their target receptor on the cell. Antibody binding can occur at different sites on the surface protein leading to various mechanisms that cause the same effect. In the case of SARS-CoV-2, two viral neutralization mechanisms by antibodies have been observed [1, 48] and shown in Fig. 1c, d. One of the mechanisms involves direct binding of antibodies to the attachment site of the SARS-CoV-2-RBD, resulting in the antibody competing with the target receptor hACE2. Another mechanism involves the binding of antibodies to the other sites on RBD without any competition with the target receptor. The latter is shown to be involved in neutralization by the most potent Monoclonal Antibody (mAb) discovered in the study [1, 48]. Analogous to the antibodies interaction with SARS-CoV-2 RBD, inhibiting the latter to engage with hACE2, a similar molecular-level model is prepared for nano-clays resulting in a similar inhibition of the coronaviruses and shown in Fig. 8. Owing to their very high affinity, nano-clays would get attracted to spikes of SARS-CoV-2 and thus restrict engagement of RBDs of these spikes with hACE2.

## Proposed Nano-clay Administration Methodology

Clay use as drug carriers has been tested multiple times, yielding promising results of little to no cytotoxicity to cells of the human body. Kaolinite clay mineral was tested for use in a potential drug delivery system and was shown to have high biocompatibility and very low cytotoxicity [11]. Poly (d,l-lactide-co-glycolide)/montmorillonite nanoparticle cytotoxicity in vitro was also demonstrated as negligible [14]. Palygorskite-polyethyleneimine-fluorescein isothiocyanate nanocomposites also exhibited almost no cytotoxicity in vitro [13]. Authors have also experienced injecting nano-clays subcutaneously for the treatment of melanoma during in vivo studies [34]. Based on the use of clay as a cancer drug carrier and in other sustained-release medicine [25–28], we propose that nano-clays may be injected as “clay-alone” medicine subject to the verification in vivo and clinical trials.

Although nano-clays are non-biodegradable, a comprehensive understanding of the design of the similar inorganic nanoparticles with their metabolic performance in the body carried out in the study [49] could also categorize these nano-clays as human body clearable inorganic agents.

## Conclusions and Recommendations

Based on all the current and past research by the authors, establishing the high-affinity potential of nano-clays, these could be mimicked as antibodies and can thus attract and engulf coronaviruses before they get engaged with human hACE2.

The results of the molecular-level simulations for the interaction of SARS-CoV-2 spike S with the clay crystallites result in the formation of closely interacting strong van der Waals attraction fields. These van der Waals attraction fields create higher CED of the clay/SARS-CoV-2 configuration. Substantial increase in total CED after addition of clay crystallites is also a testimony of a very high affinity of SARS-CoV-2 with these particles as compared to the affinity of the former with hACE2.

We propose to continue the research by carrying out in vitro interaction studies between SARS-CoV-2 and different percentage of nano-clays. Based on the optimum dose of nano-clay developed in the in vitro phase, we suggest to carry out in vivo studies on the animals. The animal study should be carried out both with and without nano-clay to finalize the nano-clay dose and should lay the foundation for the clinical trials.

## Data Availability

All data generated or analyzed during this study are included in this published article.
